# Characterization of Cry4Aa Toxin from *Bacillus thuringiensis* JW-1 and Its Insecticidal Activity Against *Bradysia difformis*

**DOI:** 10.3390/insects16121228

**Published:** 2025-12-03

**Authors:** Ping Xu, Shaoxuan Qu, Jinsheng Lin, Huiping Li, Lijuan Hou, Ning Jiang, Lin Ma

**Affiliations:** Jiangsu Key Laboratory for Horticultural Crop Genetic Improvement, Institute of Vegetable Crops, Jiangsu Academy of Agricultural Sciences, Nanjing 210014, China; pingxu@jaas.ac.cn (P.X.); ququzhibao@163.com (S.Q.); 20020005@jaas.ac.cn (J.L.); lhp211@163.com (H.L.); mybailinggu@126.com (L.H.); 19980012@jaas.ac.cn (N.J.)

**Keywords:** *Bradysia difformis*, *Bacillus thuringiensis* JW-1, Cry4Aa, insecticidal activity, biological control

## Abstract

*Bradysia difformis* is a notorious pest in mushroom production in China. Biological control using *Bacillus thuringiensis* (Bt) offers an environmentally friendly and effective strategy against this pest. Here, we show that the complete genome of strain JW-1 consists of one circular chromosome and seven circular plasmids. JW-1-Plasmid 4 comprises 127,921 bp with a GC content of 33.9%, and is predicted to contain 131 genes, including six insecticidal genes: *cry4Aa*, *cry4Ba*, *cry10Ab*, *cry11Aa*, *cyt1Aa*, and *cyt2Ba*. A 3542-bp fragment containing the *cry4Aa* gene was amplified from this strain. Phylogenetic analysis based on Cry4 toxin sequences showed that JW-1 Cry4 toxin belongs to the Cry4Aa toxin cluster. A Cry4Aa fusion protein was subsequently expressed in *E. coli* and purified using Ni-IDA affinity chromatography. A larval feeding assay showed that purified Cry4Aa was toxic to *B. difformis* larvae, with an LC_50_ of 2.71 ng/mL. These results confirmed the identity and bioactivity of Cry4Aa from strain JW-1, offering a promising biological control agent against this major pest.

## Simple Summary

Mushroom production is an important agricultural industry, but it often suffers from damage caused by the fungus gnat *Bradysia difformis*. These pests can destroy the growing mushrooms and cause significant economic losses for farmers. In this study, we investigated a natural bacterium that can control these harmful insects. We carefully analyzed the bacterium’s genetic material and found a special gene that produces a protein toxic to fungus gnat larvae. We produced this protein in a safe laboratory bacterium and tested its effect on the insects. The results showed that the protein killed most of the larvae at a very low dose, proving it is highly effective. This discovery suggests that this natural protein could be developed into a safe and environmentally friendly method to protect mushroom crops. Using such biological control methods can reduce the need for chemical pesticides, helping farmers maintain healthy production while protecting the environment.

## 1. Introduction

China is one of the largest producers and exporters of wild edible fungi in the world [1], producing over 70% of the total global output [2]. However, owing to the nutrient-rich nature of the substrate, the presence of mycelia and fruiting bodies, and the dark and humid cultivation environment, edible mushroom production is prone to infestations by pests, including fungus gnats and mites, during the cultivation period [3]. Diptera are a diverse and widely distributed order of insects, representing dominant pests of cultivated and wild edible and medicinal mushrooms worldwide. To date, over 100 species from 20 Diptera families have been documented as pests of edible mushrooms in China. Of these, the families Sciaridae, Phoridae, and Drosophilidae each contain more than 15 harmful species.

*Bradysia difformis,* a member of the Sciaridae family, is widely recognized as a major pest in greenhouse agriculture, causing substantial economic damage globally [4]. This gnat species poses a threat not only to mushroom cultivation but also to ornamental plants [5,6]. Control measures for *B*. *difformis* in edible mushroom cultivation mainly include agricultural, physical, chemical, and biological methods. During the cultivation process, strict management of substrate sterilization and environmental hygiene represents the primary agricultural strategy for the effective prevention of *B*. *difformis* infestations [3]. Meanwhile, “one net, one light, one board, and one buffer” has been proposed as a method for the physical control of *B*. *difformis* infestations in edible mushroom cultivation facilities, and has demonstrated marked efficacy against pests from the Diptera, Lepidoptera, and Coleoptera orders [7]. Compared to other control methods, chemical control offers the advantages of rapid, effective, and convenient pest damage reduction. However, the use of insecticides alone cannot provide long-term pest management and should only be regarded as a supplementary measure to other control methods [8]. *Bacillus thuringiensis* (Bt) is one of the most widely used and effective biological control methods currently available and remains a key focus of research in this field [9].

Bt is the most successful insect pathogen used for the control of insect pests, exerting its toxic effects on insect larvae through the formation of parasporal crystals with insecticidal properties [10,11]. Bt has been widely employed in the control of a broad spectrum of pests, including those belonging to the orders Lepidoptera, Diptera, and Coleoptera [12,13,14]. During sporulation, Bt produces parasporal crystals composed of endotoxins encoded by *cry* and *cyt* genes. These proteins must be ingested by insects to exert their effects, ultimately leading to mortality. Following ingestion, the alkaline conditions within the insect gut lead to the dissolution of the crystals, converting them into toxic core fragments [15].

In this study, we provide the complete sequence and annotation of the genome of JW-1. Strain JW-1 was found to harbor the *cry4Aa* gene, which was subsequently expressed in *Escherichia coli*. The purified protein product of *cry4Aa* exhibited effective insecticidal activity against *B. difformis*, demonstrating the strain’s potential as a biological control agent for this pest in mushroom cultivation.

## 2. Materials and Methods

### 2.1. Bacterial Strains, Plasmids, and Culture Conditions

The Bt strain JW-1 was obtained from soil samples collected at the Purple Mountain of Nanjing City, Jiangsu Province, China (GPS coordinates: N 118°86′16.94″; E 32°07′44.11″). For evaluating insecticidal activity, the Bt strains were cultured in beef extract-peptone-glucose (BPG) broth (beef extract 3 g/L, peptone 5 g/L, glucose 10 g/L) for 72 h at 30 °C with shaking at 220 rpm. *E. coli* cultures were propagated in Luria–Bertani (LB) broth at 37 °C (220 rpm).

### 2.2. Scanning Electron Microscopy (SEM) and Culture Conditions

Strains confirmed as Bt through molecular analysis were streaked onto culture plates and incubated at 30 °C for 2–3 days until stable sporulation was achieved. A single colony was then inoculated into LB liquid medium and cultured in a shaking incubator (220 rpm) at 37 °C for 12 h. Subsequently, 1 mL of the bacterial suspension was centrifuged at 3000 rpm for 2 min, and the resulting pellet was fixed in 1 mL of fixative solution for SEM imaging. The JW-1 strain was grown overnight at 30 °C on LB and PDA media.

### 2.3. Genome Sequencing and Assembly

Sequencing of the genome of strain JW-1 was performed by Genepioneer Biotechnologies using the Illumina HiSeq platform in combination with third-generation sequencing technology. Raw single-molecule sequencing data were quality-filtered, with the length distribution of all obtained reads serving as the main quality control metric. Illumina sequencing data were initially assembled using ABySS v2.0.2, followed by alignment of PacBio sequencing data using BLASR. Based on the alignment results, error correction was applied to the single-molecule sequencing data to minimize errors such as single-base substitutions, insertions, and deletions in long reads. The corrected data were then used for assembly, following an overlap-layout-concensus approach, similar to first-generation sequencing principles. CANU 2.2 software (https://github.com/marbl/canu) version was used for assembly [16]. After scaffold construction, Illumina data were used for validation and gap closing, ensuring quality control of the obtained sequencing data, and enabling the generation of the final genome map of strain JW-1 through bioinformatics analysis.

### 2.4. Cry4Aa Gene Analysis

A single colony of Bt strain JW-1 was cultured overnight in 5 mL of LB broth at 30 °C with shaking at 200 rpm. The bacterial cells were resuspended in 600 μL of sorbitol buffer (1.2 M sorbitol with 0.1 M phosphate-buffered saline [PBS]) containing 5 μL of lysozyme (0.05 g/mL), and then incubated overnight at 30 °C in a water bath. Total DNA was extracted from JW-1 using the Bacterial Genome Extraction Kit from TsingKe (Nanjing, China). The primers used to amplify the full *cry4Aa* gene of JW-1 (JW-1-*Cry4Aa*F: 5′-AAGCACGCCATATCGCCGAAAGGCACA-3′, JW-1-*Cry4Aa*R: 5′-GGCAGGGATCTTAGATTCTGTGCTTT-3′) were designed based on the complete *cry4Aa* gene of strain JW-1-Plasmid 4 (GenBank Accession No. QFP86699.1) using Primer Premier 5 software [17]. PCR amplification was performed using I-5 Hi-Fi DNA Polymerase (TsingKe Company). The cycling conditions were 35 cycles of 98 °C for 10 s, 53 °C for 20 s, and 72 °C for 90 s. The definitive list of Bt toxins are found in the Bacterial Pesticidal Protein Resource Center (www.bpprc.org). That lists eight different Cry4 sequences, those are used in phylogenetic tree was constructed based on the Cry4Aa toxin sequence of JW-1 using MEGA 5.0 software [18]. The evolutionary history of JW-1 was inferred using the maximum likelihood method with a bootstrap analysis with 1000 replications [19].

### 2.5. Expression Vector Construction

The full-length PCR product of *cry4Aa* was inserted between the NdeI and XbaI sites of the pCzn1 vector. The resulting recombinant plasmid, pCzn1-*cry4Aa*, was transformed into Top10 competent cells and identified on LB agar supplemented with ampicillin (100 μg/mL). The presence of the *cry4Aa* gene in *E. coli* colonies was confirmed using primers JW-1-*Cry4Aa*F/R.

The expression of Cry4Aa was induced by adding 0.5 mM isopropyl β-D-1-thiogalactopyranoside (IPTG) to the culture, followed by overnight incubation at 20 °C. After induction, the bacterial solution (1 mL) was centrifuged at 10,000 rpm for 2 min, and the resulting pellet was resuspended in 1× loading buffer (20 mg/mL SDS, 0.1 mg/mL bromophenol blue, 1 M Tris-HCl, pH 6.8 [25 μL/mL], 0.1 mL/mL glycerol; 5 μL/mL β-mercaptoethanol was added before use). After sonication to disrupt the cells, the suspension was centrifuged at 4000 rpm for 10 min, the resulting pellet was resuspended in PBS, and proteins in the supernatant and precipitate were analyzed using sodium dodecyl sulfate–polyacrylamide gel electrophoresis (SDS-PAGE).

### 2.6. Purification of the Cry4Aa Fusion Protein

The fusion protein was purified as previously reported [20]. In brief, the supernatant obtained from the Cry4Aa preparation was loaded onto a Ni-IDA Sepharose CL-6B affinity chromatography column, which had been pre-equilibrated with Ni-IDA binding buffer, at a flow rate of 0.5 mL/min. The column was sequentially washed with Ni-IDA binding buffer at 0.5 mL/min and Ni-IDA washing buffer (20 mM Tris–HCl, pH 8.0, containing 20 mM imidazole and 150 mM NaCl) at 1 mL/min until the OD280 of the effluent reached baseline levels. The target protein was eluted using Ni-IDA elution buffer (20 mM Tris–HCl, pH 8.0, containing 250 mM imidazole and 150 mM NaCl) at 1 mL/min. The collected protein solution was dialyzed overnight against Tris-buffered saline (TBS) (20 mM Tris–HCl, 150 mM NaCl, pH 8.0) and subsequently analyzed by 12% SDS-PAGE.

### 2.7. Western Blot

After purification, the recombinant Cry4Aa protein was separated via SDS-PAGE and subsequently transferred onto a PVDF membrane. The membrane was then washed four times with PBS containing 0.1% Tween 20 (PBST) and incubated overnight at 4 °C with a primary anti-His-tag antibody (Zoonbio Biotechnology, Nanjing, China) diluted in 5% skimmed milk blocking buffer (1:1000). After three washes with PBST, the membrane was incubated at 37 °C for 1 h with alkaline phosphatase-conjugated goat anti-mouse IgG (Zoonbio Biotechnology) diluted 1:5000 in 5% skimmed milk blocking buffer, and then washed again four times with PBST. Protein bands were visualized using enhanced chemiluminescence (Figure S1). The multitag protein is a single polypeptide chain harboring twenty-five distinct epitope or affinity tags arranged in tandem (His, T7, H5V, Glu-Glu, V5, F-tag, TC-tag, Avi-tag, Spftag-1, Spftag-3, Xpress, Spy-tag, Snoop-tag, 3×FLAG, Hxv, S-tag, calmodulin-tag, 5BP-tag, KT3, F2, AU1, AU5, Axi-tag, GST and HA). Because the multitag protein includes an internal His epitope, it is recognized by the identical anti-His antibody employed for the target protein and thus functions as an on-blot positive control for antibody specificity and assay sensitivity.

### 2.8. Insecticidal Activity Test

The third-instar larvae of *B difformis* were used as the experimental subjects to assess the toxicity of the Cry4Aa insecticidal protein from strain JW-1. The Cry4Aa protein was extracted from the JW-1 strain, and fresh fruiting bodies of Auricularia polytricha (with a diameter of 1 cm) were immersed in 2 mL of the Cry4Aa insecticidal protein solution. The treated fruiting bodies were then placed on 9 cm petri dishes for further analysis. A filter paper (Sangon Biotech, Shanghai, China) soaked with 0.8 mL of the respective solutions was placed at the bottom of each plate. Thirty larvae were transferred to each plate and incubated at 25 ± 1 °C with 65 ± 5% relative humidity for 72 h. All experiments were conducted in triplicate. The sterile water was used as a positive control. The mortality and corrected mortality rates of *B difformis* larvae were determined at various concentrations of Cry4Aa protein, including 0.1 ng/mL, 0.5 ng/mL, 1 ng/mL, 5 ng/mL, 10 ng/mL, and 100 ng/mL [21]. Statistical analysis was performed on the data obtained from these treatments.

## 3. Results

### 3.1. Morphological Identification

**Figure 1:**
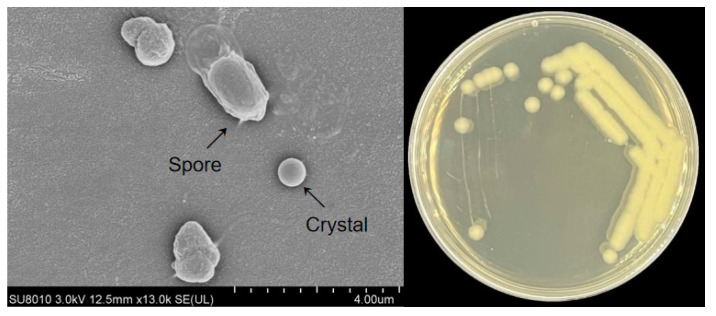
(**Left**): scanning electron micrograph of Bt spores. (**Right**): colony morphology after 24 h of growth on LB medium.

The spore morphology of strain JW-1 was examined by SEM (Figure 1 left). Strain JW-1 is a Gram-positive bacterium that produces parasporal crystals during sporulation. The spores are approximately 1–1.5 µm in diameter and can reach 2–4 µm in length. During the early growth stage, Bt spores exhibited a rounded shape. However, as culture progressed, they became flattened, developed radial wrinkles on the surface, lost their glossiness, and displayed more pronounced irregular edges. Some spores and crystals were undergoing separation, and invaginations were observed in the cell membrane. During sporulation, parasporal crystals of various shapes form within the cells. The spores are oval, whereas the parasporal crystals are smaller and appear as either spherical or irregular inclusions. Strain JW-1 exhibited a moderate growth rate, producing colonies of approximately 2 mm in diameter after overnight incubation at 30 °C (Figure 1 right). On LB agar, the colonies were white, opaque, and shiny. In addition, JW-1 was capable of growing on other media, including PDA.

### 3.2. Genome Properties

**Figure 2:**
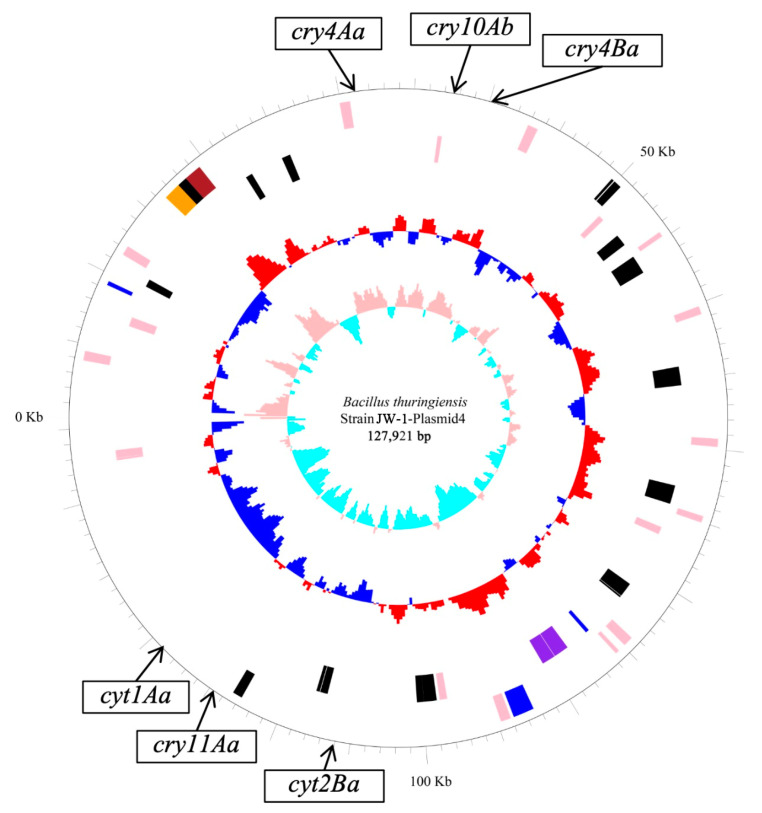
Circular representation of JW-1-Plasmid4. Rings from outer to inner: The outermost circle represents the genome size (0.5 Mb per tick); the second and third circles show coding sequences on the forward and reverse strands, with different colors indicating COG functional categories; the fourth circle represents rRNA and tRNA; the fifth circle shows the GC content; and the innermost circle indicates the GC skew.

The complete genome of strain JW-1 consists of one circular chromosome and seven circular plasmids (Plasmid 1–7). The circular chromosome is 5,500,376 bp in size, has a GC content of 32.326%, and is predicted to contain 5645 genes. The gene prediction results, including the sizes of genes, the number of genes, total gene length, GC content within coding regions, and GenBank accession details, are presented in Table 1. Genomic analysis revealed that all insecticidal genes are located in JW-1-Plasmid 4. This plasmid has a length of 127,921 bp with a GC content of 33.9%, and is predicted to harbor 131 genes, including 6 insecticidal genes: *cry4Aa*, *cry4Ba*, *cry10Ab*, *cry11Aa*, *cyt1Aa*, and *cyt2Ba*. Additionally, Plasmid 4 harbors genes encoding crystal proteins and other functionally important proteins. The complete sequence of the genome of strain JW-1, together with its annotation, has been deposited in GenBank under the Accession No. (PRJNA574201). Circular representation of JW-1-Plasmid4 is shown in Figure 2.

### 3.3. PCR Amplification of cry4 Genes

**Figure 3:**
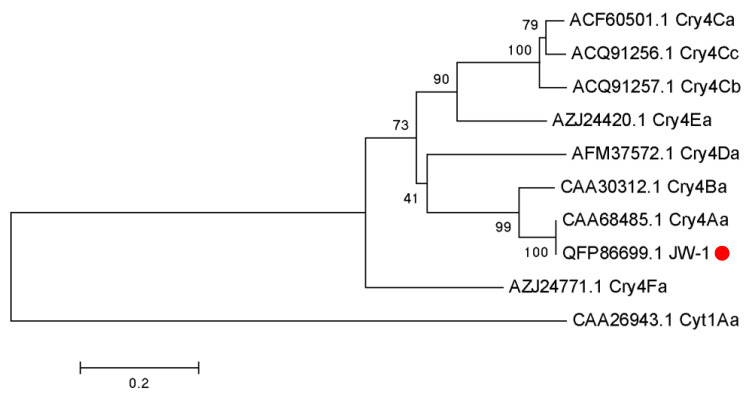
Phylogenetic tree based on Cry4 toxin sequences. Genetic distances were calculated using the Jones–Taylor–Thornton model, and dendrograms were generated using the maximum likelihood method. The initial tree for the heuristic search was automatically obtained using the neighbor-joining approach. Exhibited 100% identity to the reported Cry4Aa toxin, confirming that this protein is a member of Cry4Aa in the Cry4 family.

PCR amplification of *cry4* genes from the genomic DNA of Bt strain JW-1 yielded a 3542-bp fragment corresponding to the *cry4Aa* gene. The sequence of the resulting Cry4 toxin shared 100% identity with the known Cry4Aa protein, confirming that it belonged to the Cry4Aa toxin family (Figure 3).

### 3.4. Expression Vector Assembly

**Figure 4:**
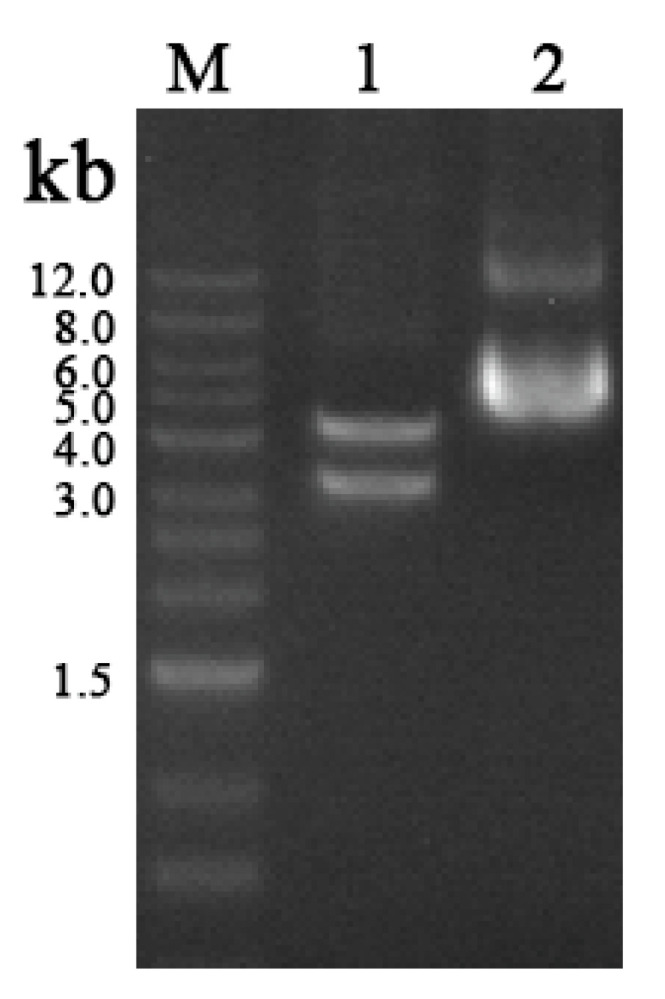
Digestion of the pCzn1-*cry4Aa* vector using the restriction endonucleases NdeI and XbaI resulted in fragments of approximately 3.5 and 4 kb (lane 1). The original pCzn1-*cry4Aa* plasmid was approximately 7.5 kb in length (lane 2).

The full-length *cry4Aa* gene was inserted into the pCzn1 vector via seamless cloning, using the NdeI and XbaI restriction enzymes for double digestion. Digestion analysis yielded two distinct bands, one of approximately 3.5 kb, corresponding to the full-length *cry4Aa* gene, and the other comprising 4 kb, corresponding to the vector backbone (Figure 4).

### 3.5. Stimulated Expression

**Figure 5:**
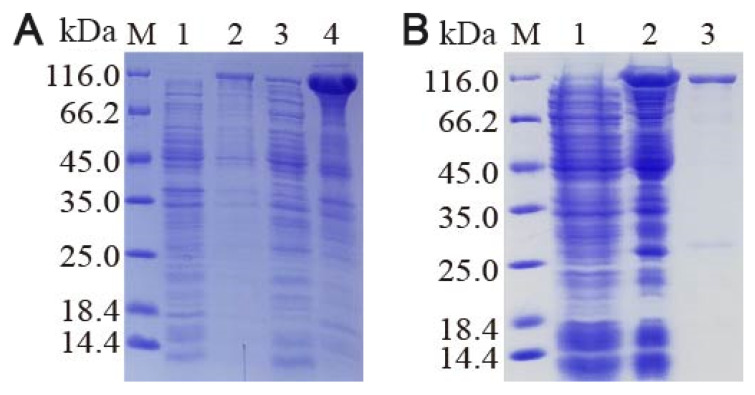
Expression and purification of the Cry4Aa fusion protein as determined by SDS-PAGE. (**A**) Protein expression before IPTG induction (lane 1), after IPTG induction (lane 2), in the supernatant following IPTG-induced sonication (lane 3), and in the precipitate following IPTG-induced sonication (lane 4). (**B**) Protein sample after cell sonication (lane 1), in the column effluent (lane 2), and in the eluted fraction (lane 3).

The expression of Cry4Aa toxin in *E. coli* was confirmed by SDS-PAGE (Figure 5A). Furthermore, Cry4Aa was present in the supernatant primarily in a soluble form and could be directly purified by affinity chromatography.

### 3.6. Recombinant Protein Isolation and Western Blot Confirmation

**Figure 6:**
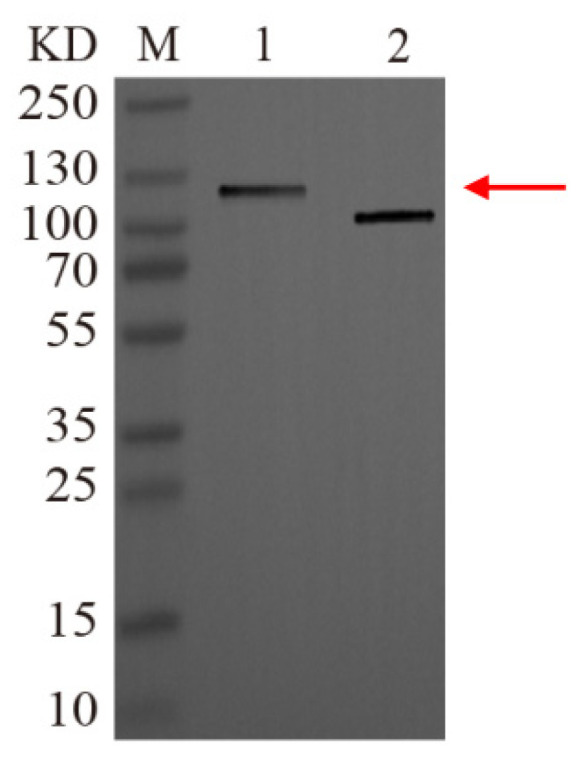
Western blot analysis of purified recombinant Cry4Aa. Cry4Aa protein (lane 1), Multitag protein (lane 2). The red arrow points to the Cry4Aa protein.

Following purification of the fusion protein by Ni-affinity chromatography, the initial bacterial homogenate, column effluent, and eluate were separately analyzed using 12% SDS-PAGE. The analysis showed that the target protein was enriched in the eluted fractions in a relatively pure form (Figure 5B). The purified protein was subsequently subjected to western blotting (Figure 6), which confirmed the soluble nature of the recombinant protein.

### 3.7. Bioassay-Based Analysis of Cry4Aa Toxin Activity

**Figure 7:**
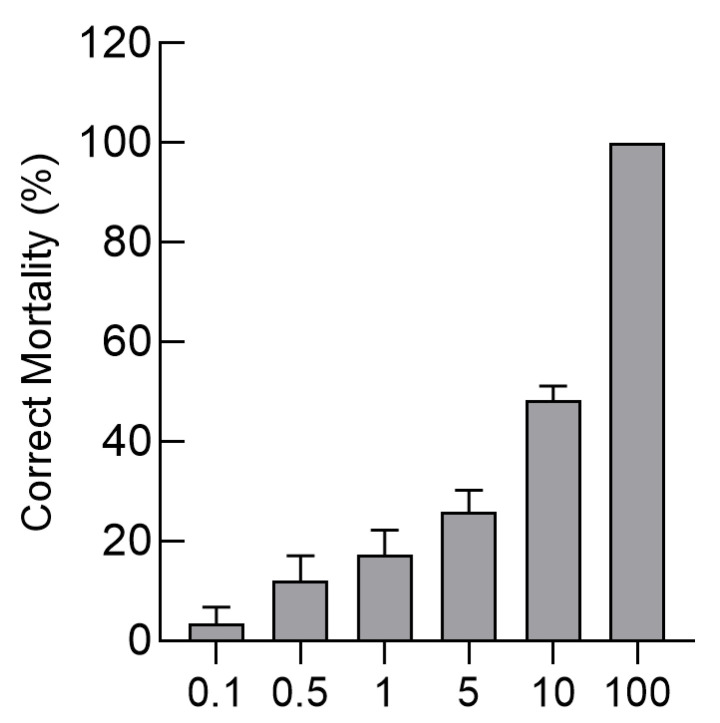
The toxicity of purified Cry4Aa in *B. difformis* larvae. Bars represent corrected mortality (%) at different protein concentrations: 0.1, 0.5, 1, 5, 10, and 100 ng/mL. An oral ingestion method was employed for in vitro toxicity assessment. After 72 h of exposure, the insecticidal toxicity of Cry4Aa toxin against third-instar *B. difformis* larvae was analyzed at five different protein concentrations.

Bioassays were conducted on third instar larvae of *B. difformis* to evaluate the insecticidal activity of the Cry4Aa toxin. After 72 h of exposure to the toxin, larval mortality exhibited a dose-dependent response, and there was a linear correlation between the logarithm of the toxin concentration and the probit-transformed mortality rate. The regression equation was determined to be *y* = 4.20 + 1.84*x*, where *x* represents the log-transformed concentration and *y* the probit value of mortality. The median lethal concentration (LC_50_) of Cry4Aa was calculated as 2.71 ng/mL, with 95% fiducial limits ranging from 1.87 to 3.92 ng/mL. The regression coefficient (*R*) for the model was 0.8881, indicating a strong fit to the probit model. As shown in (Figure 7), at Cry4Aa protein concentrations of 0.1 ng/mL, 0.5 ng/mL, 1 ng/mL, 5 ng/mL, 10 ng/mL, and 100 ng/mL, the corrected mortality rates were 3.45 ± 3.33%, 12.07 ± 5.00%, 17.24 ± 5.00%, 25.86 ± 4.41%, 48.28 ± 2.89%, and 100%, respectively. After treatment with Cry4Aa for 72 h, most larvae exhibited darkening or began to disintegrate.

## 4. Discussion

We have previously found that the Cyt2 toxin from JW-1, a recently identified strain of Bt, exhibits significant insecticidal activity against *B. difformis* [21]. Additionally, this strain carries a Cry4Aa-encoding gene, which also demonstrates excellent insecticidal efficacy against this pest. However, the insecticidal potential of this toxin protein (Cry4Aa) has not been thoroughly evaluated and is thus the focus of the present study.

Comparative genomic analysis revealed that plasmid 4 of strain JW-1 shows remarkable similarity to the well-characterized pBtoxis plasmid of *Bacillus thuringiensis israelensis* (Bti). Both plasmids are comparable in size (JW-1 plasmid 4: 127,921 bp; pBtoxis: ~128 kb) and GC content (~33–34%), and share an almost identical insecticidal gene composition, including *cry4Aa, cry4Ba, cry10Ab, cry11Aa, cyt1Aa*, and *cyt2Ba* [22]. A BLAST +2.17.0 comparison indicated over 99.9% nucleotide identity between JW-1 plasmid 4 (GenBank: CP045026.1) and pBtoxis, suggesting a very close evolutionary relationship. These findings indicate that JW-1 is phylogenetically affiliated with the *B. thuringiensis israelensis* group and likely represents a new environmental isolate of Bti rather than a genetically distinct species. Nevertheless, JW-1 possesses its own genomic configuration, with differences in the number and composition of auxiliary plasmids, gene content outside plasmid 4, and potentially in ecological origin or insecticidal activity. Therefore, while JW-1 shares a nearly identical toxin plasmid with classical Bti strains, it remains of interest as a naturally occurring isolate that may offer insights into the genomic diversity and evolution of Bti-like entomopathogenic bacteria.

Cry insecticidal proteins exhibit specific toxicity against Lepidoptera, Diptera, Coleoptera, Hymenoptera, and nematodes [23,24]. Different Cry proteins have distinct insecticidal spectra, with a small subset capable of targeting two or three insect classes [25]. Currently, there are approximately 760 known Cry protein variants [26]. Cry4Aa, a three-domain Cry toxin produced by *Bacillus thuringiensis* subsp. *israelensis*, exhibits insecticidal activity against various mosquito species [27,28]. Its crystal structure has been determined, revealing distinct functional roles for each domain. Domain I mediates pore formation in the insect midgut [28,29,30,31,32], Domain II participates in receptor binding [28,33,34,35], and Domain III contributes to both receptor interaction and structural stability [28,35,36,37]. Initially synthesized as a 130-kDa protoxin, Cry4Aa undergoes proteolytic processing to yield 45 and 20 kDa protease-resistant fragments via a 60–65 kDa intermediate. These fragments, generated through intramolecular cleavage, reassemble via electrostatic interactions, forming the functional toxin monomer, with both fragments essential for toxicity [28,38]. Computational modeling suggests that three toxin monomers oligomerize through Domain I, forming a trimeric transmembrane pore structure involving specific helical regions of Domain I [39].

In this study, Cry4Aa, produced by *Bacillus thuringiensis*, is a dipteran-specific toxin with considerable potential for mosquito control. However, its relatively low expression in *E. coli* remains a major limitation for large-scale bioinsecticide production. Codon optimization to match codon usage in *E. coli* led to efficient production of Cry4Aa [40]. Furthermore, enhancing toxicity by combining Cry4Aa and Cry11Ba represents a promising strategy for mosquito control efforts [41]. Enhanced expression of the polyphosphate kinase (*ppk*) gene has also been linked to an increase in bioinsecticide synthesis in Bt [42]. Other strategies, such as domain swapping, site-directed mutagenesis, and peptide addition, have been employed to enhance the toxicity of Bt toxins against specific insects [43]. Future research efforts will focus on the development of highly efficient insecticidal strains and further characterizing the insecticidal activities of crystal proteins from strain JW-1.

## Figures and Tables

**Table 1:** Statistics for the genome of strain JW-1.

	Bases (bp)	Gene Number	Gene Total Length (bp)	GC Content in Coding Regions (%)	GenBank Accession No.
JW-1-chromosome	5,500,376	5645	4,582,035	35.8	CP045030.1
JW-1-Plasmid1	359,606	340	262,059	33.5	CP045023.1
JW-1-Plasmid 2	349,211	449	300,957	33.7	CP045024.1
JW-1-Plasmid 3	235,425	247	208,911	36.6	CP045025.1
JW-1-Plasmid 4	127,921	131	94,935	33.9	CP045026.1
JW-1-Plasmid 5	14,935	28	13,209	39.8	CP045027.1
JW-1-Plasmid 6	6824	3	2292	31.8	CP045028.1
JW-1-Plasmid 7	4974	3	3045	34.5	CP045029.1

## Data Availability

The original contributions presented in this study are included in the article/Supplementary Materials. Further inquiries can be directed to the corresponding author.

## Supplementary Materials

The following supporting information can be downloaded at: https://www.mdpi.com/article/10.3390/insects16121228/s1, Figure S1: Uncropped images for protein gels, related to Figure 6.
