# Supplementary material for: Characterization of Cry4Aa Toxin from Bacillus thuringiensis JW-1 and Its Insecticidal Activity Against Bradysia difformis
Source: Insects. 2025 Dec 3;16(12):1228. doi: 10.3390/insects16121228 (PMC12734002; doi:10.3390/insects16121228)
Supplement: Supplementary file 1 [file insects-16-01228-s001.zip › insects-3921908-supplementary.pdf]

Supplementary data

Characterization of Cry4Aa Toxin from *Bacillus thuringiensis* JW-1 and Its Insecticidal Activity Against *Bradysia difformis*

Ping Xu, Shaoxuan Qu, Jinsheng Lin, Huiping Li, Lijuan Hou, Ning Jiang and Lin Ma *

Jiangsu Key Laboratory for Horticultural Crop Genetic Improvement, Institute of Vegetable Crops, Jiangsu Academy of Agricultural Sciences, Nanjing 210014

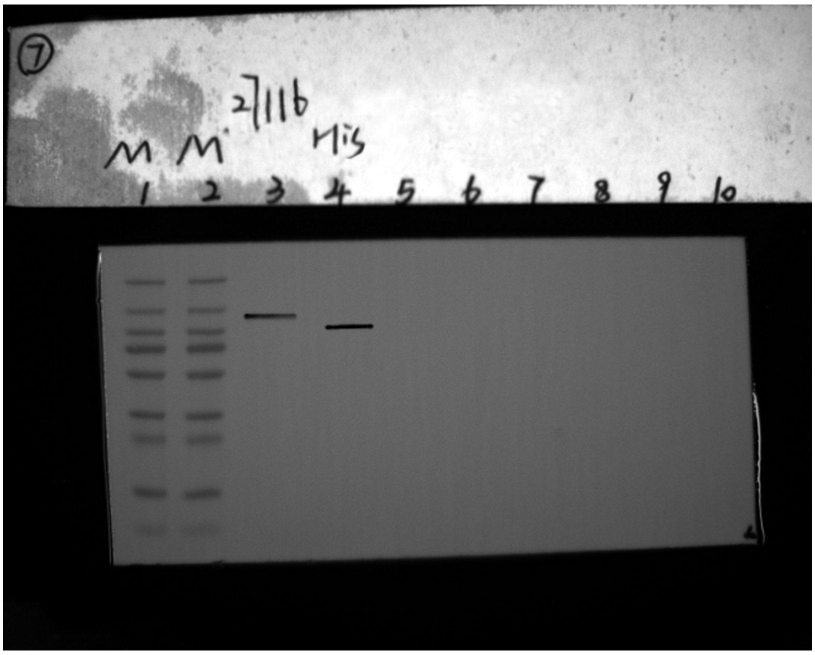

Figure S1. Uncropped images for protein gels, related to Figure 6.
